# A retrospective analysis in patients with EGFR-mutant lung adenocarcinoma: is EGFR mutation associated with a higher incidence of brain metastasis?

**DOI:** 10.18632/oncotarget.10933

**Published:** 2016-07-29

**Authors:** Guang Han, Jianping Bi, Wenyong Tan, Xueyan Wei, Xiaohong Wang, Xiaofang Ying, Xiaofang Guo, Xiaoyi Zhou, Desheng Hu, Weining Zhen

**Affiliations:** ^1^ Department of Radiation Oncology, Hubei Cancer Hospital, Wuhan, HB, China; ^2^ Department of Oncology, Shenzhen People Hospital, Shenzhen, China; ^3^ Department of Radiology, Hubei Cancer Hospital, Wuhan, HB, China; ^4^ Department of Radiation Oncology, University of Nebraska Medical Center, Omaha, NE, USA

**Keywords:** EGFR, mutation, brain metastasis, lung adenocarcinoma, retrospective study

## Abstract

Lung adenocarcinomas are more commonly associated with brain metastases (BM). Epidermal growth factor receptor (EGFR) mutations have been demonstrated to be both predictive and prognostic for patients with lung adenocarcinoma. We aimed to explore the potential association between EGFR mutation and the risk of BM in pulmonary adenocarcinoma patients. Data of 234 patients from 2007 to 2014 were retrospectively reviewed. A total of 108 patients had EGFR mutations in the entire cohort. Among them, 76 patients developed BM during their disease course. The incidence of BM was statistically higher in patients with EGFR mutations both at initial diagnosis (*P*=0.014) and at last follow-up (*P*<0.001). Multivariate logistic regression analysis revealed that EGFR mutation significantly increased the risk of BM at initial diagnosis (OR=2.515, *P*=0.022). In patients without BM at initial diagnosis, the accumulative rate of subsequent BM was significantly higher with EGFR mutations (*P*=0.001). Multivariate Cox regression analysis identified EGFR mutation as the only independent risk factor for subsequent BM (HR=3.036, *P*=0.001). Patients with EGFR mutations demonstrated longer overall survival (OS) after BM diagnosis than patients with wild-type EGFR (*P*=0.028). Our data suggest that EGFR mutation is an independent predictive and prognostic risk factor for BM and a positive predictive factor for OS in patients with BM.

## INTRODUCTION

Approximately 80% of lung cancer cases have been classified as non-small cell lung carcinoma (NSCLC), which can be further divided into subtypes according to specific histology such as adenocarcinoma, squamous cell carcinoma, and large cell carcinoma [1]. Among these pathological subtypes, adenocarcinoma is the most common subtype; which occurs in more than half of NSCLC patients. Furthermore, adenocarcinoma is more aggressive than other NSCLC subtypes, and is often associated with rapid disease progression and early distant metastasis [2, 3]. Brain metastases (BM) develop in 22-54% of NSCLC patients during the disease course. Studies have shown that the incidence of BM is higher with adenocarcinoma than with the other subtypes of NSCLC [3, 4]. Approximately 45-52% of patients with lung adenocarcinoma develop BM at some point in their disease course, which compares unfavorably with a less than 20% incidence of BM for squamous cell carcinoma [5, 6]. In general, the prognosis for patients with BM remains poor, with a median overall survival (OS) of 2-3 months when treated with systemic corticosteroid alone, and a median OS of 3-6 months when treated with whole brain radiation therapy (WBRT) [7, 8].

Recently, several studies have reported the benefits of epidermal growth factor receptor (EGFR) tyrosine kinase inhibitors (TKIs) for NSCLC patients with BM. The median OS of patients with BM significantly improved with TKI treatment, which ranged from 11.8 to 18.8 months [9–11]. Therefore, the BM treatment in this patient population has been guided by the EGFR mutation status.

Among all subtypes of NSCLC, EGFR mutations are predominantly found in adenocarcinomas [12]. The detection of EGFR mutations sensitizing L858R and deletion 19 may warrant treatment with TKIs such as gefitinib, erlotinib and icotinib for patients with advanced pulmonary adenocarcinoma. EGFR signaling pathways in lung cancer have been reported to promote angiogenesis, cellular proliferation and epithelial-mesenchymal transition (EMT); and all of which may mediate oncogenic progression and metastasis [13–15]. The biological characteristics of EGFR may also reflect the pattern of metastasis. Studies have demonstrated an association between EGFR genetic alterations and distant metastases in patients with breast cancer [16, 17]. Furthermore, in patients with pulmonary adenocarcinomas, different pulmonary metastatic patterns in EGFR-mutated tumors have also been reported [18]. Despite these findings, a clear relationship between EGFR mutation and the BM of lung adenocarcinomas remains to be determined. Some studies have suggested that patients with EGFR mutations may have a higher incidence of BM than patients without EGFR mutations [11, 19, 20]. However, these were not supported by other studies [21, 22]. Due to the small sample size and low numbers of patients included for EGFR mutation analyses in these studies, it was not possible to fully answer this question.

The purposes of this study were to explore the potential correlation between EGFR mutation status and the risk of BM in patients with pulmonary adenocarcinoma, and to compare the OS of pulmonary adenocarcinoma patients with BM according to their EGFR mutation status.

## RESULTS

### Patient characteristics

A total of 234 patients with a median age of 57.5 years (range: 27-87 years) at initial diagnosis were consecutively enrolled into this study. Patient characteristics are summarized in Table 1. Among these patients, 76 (32.5%) had documented BM, 39 (16.7%) had initial BM, and 37 (15.8%) had subsequent BM.

**Table 1:** Baseline Characteristics (N = 234)

Characteristics	Total (%)	EGFR Mutation		*P*
		Negative (%)	Positive (%)	
Gender				<0.001
Male	125 (53.4)	81 (64.8)	44 (35.2)	
Female	109 (46.6)	45 (41.3)	64 (58.7)	
Age				0.282
<60	143 (61.1)	81 (56.6)	62 (43.4)	
≥60	91 (38.9)	45 (49.5)	46 (50.5)	
Smoking				<0.001
No	131 (56.0)	57 (43.5)	74 (56.5)	
Yes	103 (44.0)	69 (67.0)	34 (33.0)	
T				0.353
T1-2	118 (50.4)	60 (50.8)	58 (49.2)	
T3-4	116 (49.6)	66 (56.9)	50 (43.1)	
N				0.916
N0-1	81 (34.6)	44 (54.3)	37 (45.7)	
N2-3	153 (65.4)	82 (53.6)	71 (46.4)	
M				0.429
M 0	130 (55.6)	73 (56.2)	57 (43.8)	
M 1	104 (44.4)	53 (51.0)	51 (49.0)	
Clinical stage				0.300
I	18 (7.7)	8 (44.4)	10 (55.6)	
II	29 (12.4)	14 (48.3)	15 (51.7)	
III	84 (35.9)	52 (61.9)	32 (38.1)	
IV	103 (44.0)	52 (50.5)	51 (49.5)	
Initial ECMO				0.313
No	168 (71.8)	87 (51.8)	81 (48.2)	
Yes	66 (28.2)	39 (59.1)	27 (40.9)	
Final ECMO				0.494
No	144 (61.5)	75 (52.1)	69 (47.9)	
Yes	90 (38.5)	51 (56.7)	39 (43.3)	
Initial BM				0.014
No	195 (83.3)	112 (57.4)	83 (42.6)	
Yes	39 (16.7)	14 (35.9)	25 (64.1)	
Final BM				<0.001
No	158 (67.5)	98 (62.0)	60 (38.0)	
Yes	76 (32.5)	28 (36.8)	48 (63.2)	

Abbreviations: BM= brain metastases; ECMO= extracranial metastases only; EGFR= epidermal growth factor receptor; M= metastasis; N= node; T= tumor.

A total of 108 patients had EGFR mutations. Among them, 56 (51.9%) patients had exon 19 deletion, 44 (40.7%) patients had exon 21 L858R point mutation, and eight (7.4%) patients had mutations in other sites (three with exon 20 mutation, two with exon 18 mutation, and three with double mutations).

### EGFR mutation status and baseline patient characteristics

As shown in Table 1, female had a higher rate of EGFR mutation compared with male (58.7% *vs*. 35.2%, *P*<0.001). Furthermore, EGFR mutation rate was also higher in never-smokers than in smokers (56.5% *vs*. 33.0%, *P*<0.001). No statistically significant difference (*P*=0.282) in EGFR mutation rate was identified in the different age groups (below 60 years *vs*. above 60 years). There was no significant association between EGFR mutation and TNM classifications or clinical stage. In this cohort of patients, the effect of EGFR mutation on tumor metastases is site specific. Compared with patients with wild-type EGFR, patients with EGFR mutations demonstrated a higher rate of BM, regardless of whether it was initial BM (64.1% *vs*. 35.9%, *P*=0.014) or final BM (63.2% *vs*. 36.8%, *P*<0.001). However, EGFR mutation did not affect the rate of extracranial metastases only (ECMO), regardless of whether it was initial ECMO (40.9% *vs*. 59.1% [wild-type], *P*=0.313) or final ECMO (43.3% *vs*. 56.7% [wild-type], *P*=0.494).

### EGFR mutation status and characteristics of BM

**Figure 1:**
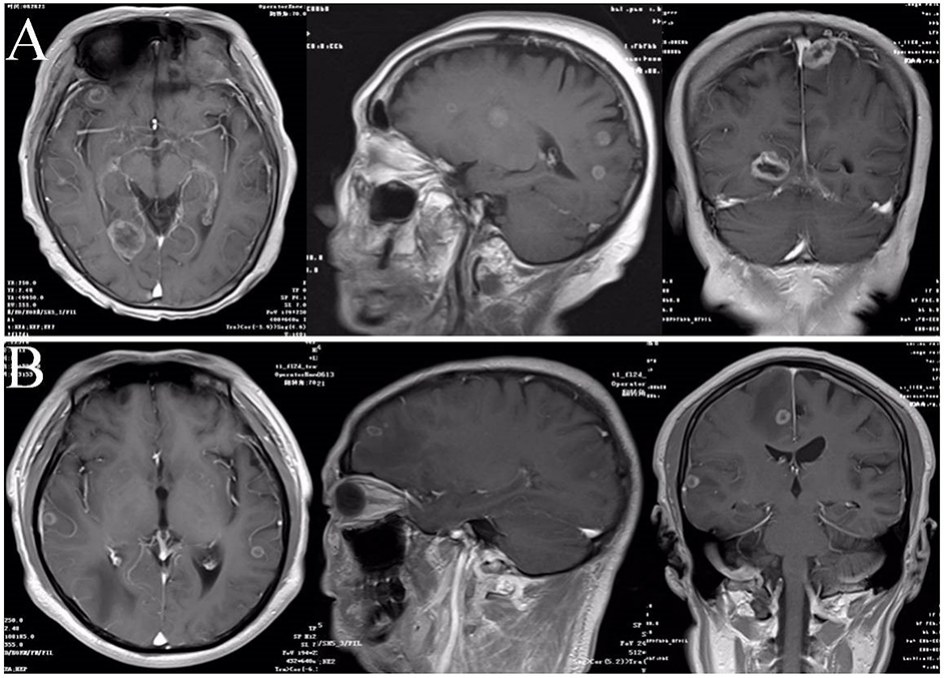
Representative results of brain MRI scans in patients with different EGFR mutation status **A.** MRI scans in patients with EGFR-mutant. **B.** MRI scans in patients with wild-type EGFR.

In order to investigate the potential correlations between the characteristics of BM and EGFR mutation, analyses were performed in patients with final BM stratified by EGFR mutation status (*n*=76). Representative results of brain magnetic resonance imaging (MRI) scans in patients with wild-type EGFR or EGFR mutations are shown in Figure 1. The numbers and sizes of the largest metastatic brain lesion between the different EGFR mutation status groups are detailed in Table 2.

**Table 2:** Characteristics of BM in patients with final BM stratified by EGFR mutation status (n=76)

Characteristics	Total (N = 76)	Wild-type EGFR (N = 28)	EGFR-mutant (N = 48)	*P*
Number of metastatic brain tumors	5.59±0.829	3.43±0.594	6.85±1.235	0.045
Size of the largest metastatic brain tumor (cm)	1.69±0.116	1.21±0.175	1.97±0.139	0.001

Abbreviations: BM= brain metastases, EGFR= epidermal growth factor receptor.

### Risk factors for initial BM

Table 3 shows the clinical factors known to be associated with initial BM. Univariate analysis revealed that female gender, no smoking history, T3-4, N2-3, and EGFR mutation were significantly associated with increased risk of BM prior to treatment (*P*<0.05). Next, each individual risk factor for initial BM was further evaluated using the multivariate logistic regression model. EGFR mutation was strongly associated with initial BM (odds ratio [OR]=2.52, 95% confidence interval [CI]=1.14-5.54, *P*=0.022). Similarly, T3-4 and N2-3 diseases were also found to be significant risk factors of initial BM (T3-4, OR=1.91, 95% CI=1.24-2.96, *P*=0.004; N2-3, OR=1.81, 95% CI=1.15–2.84, *P*=0.010), while smoking status and female gender were no longer statistically significant for being related to initial BM.

**Table 3:** Clinical features and risk factor analysis of initial BM (n=234)

Characteristics	Total (n=234)	Initial brain metastases		Univariate	Multivariate		
		BM- (n=195)	BM+ (n=39)	*P*	*P*	OR	95%CI
Gender				0.006	0.305	1.699	0.617-4.675
Male	125 (53.4)	112 (89.6)	13 (10.4)				
Female	109 (46.6)	83 (76.1)	26 (23.9)				
Age				0.436			
<60	143 (61.1)	117 (81.8)	26 (18.2)				
≥60	91 (38.9)	78 (85.7)	13 (14.3)				
EGFR Mutation Status				0.014	0.022	2.515	1.142-5.542
Negative	126 (53.8)	112 (88.9)	14 (11.1)				
Positive	108 (46.2)	83 (76.9)	25 (23.1)				
Smoking				0.011	0.178	0.477	0.163-1.401
No	131 (56.0)	102 (77.9)	29 (22.1)				
Yes	103 (44.0)	93 (90.3)	10 (9.7)				
T				0.001	0.004	1.913	1.235-2.961
T1-2	118 (50.4)	108 (91.5)	10 (8.5)				
T3-4	116 (49.6)	87 (75.0)	29 (25.0)				
N				0.002	0.010	1.810	1.154-2.839
N0-1	81 (34.6)	76 (93.8)	5 (6.2)				
N2-3	153 (65.4)	119 (77.8)	34 (22.2)				

Abbreviations: BM= brain metastases; CI= confidence interval; ECMO= extracranial metastases only; EGFR= epidermal growth factor receptor; N= node; T= tumor; OR= odd ratio.

### Risk factors for subsequent BM

**Figure 2:**
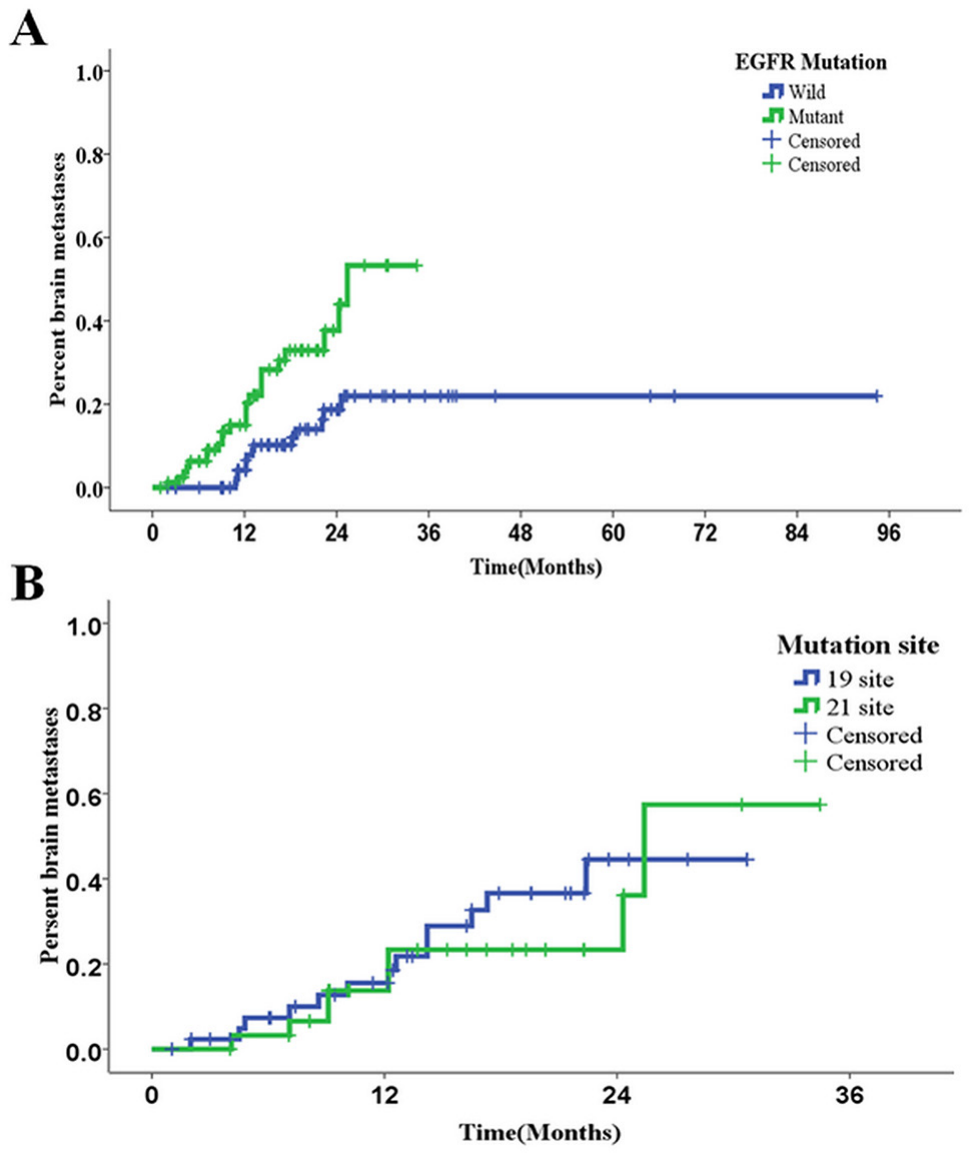
Kaplan-Meier curves for accumulative rates of subsequent brain metastasis **A.** Rates of subsequent brain metastases in patients with different EGFR mutation statuses (*P*=0.001). **B.** Rates of subsequent brain metastases in patients with different EGFR-TKI sensitive mutation sites (*P*=0.619).

To evaluate independent prognostic factors for subsequent BM, a subgroup analysis for the risk of subsequent BM was performed in patients without BM at initial diagnosis. This subgroup analysis included 195 patients without BM at initial diagnosis, with a median follow up time of 16.2 months (range: 1.0-94.4 months). Univariate analysis revealed that the female gender (*P*=0.017) and EGFR mutation (*P*=0.001) significantly increased the risk of subsequent BM. However, when further assessing these results by multivariate Cox regression analysis, only EGFR mutation was shown to be an independent risk factor for subsequent BM (hazard ratio [HR]=3.036, *P*=0.001; Table 4). In these patients, 1-, 2- and 3-year accumulative brain metastasis rates were significantly different between the wild-type EGFR group (112 patients) and EGFR-mutant group (83 patients) (4.2% [wild-type] *vs*. 15.0%, 18.7% [wild-type] *vs*. 37.7%, and 22.0% [wild-type] *vs*. 53.3%; at 1, 2 and 3 years, respectively; P=0.001) (Figure 2A).

**Table 4:** Univariate and multivariate analyses of risk factors for subsequent BM (n=195)

Characteristics	Total	Subsequent brain metastases		Univariate	Multivariate		
	(n=195)	BM- (n=158)	BM+ (n=37)	*P*	*P*	HR	95%CI
Gender				0.017	0.289		
Male	112 (57.4)	95 (84.8)	17 (15.2)				
Female	83 (42.6)	63 (75.9)	20 (24.1)				
Age				0.951	0.852		
<60	113 (57.9)	90 (79.6)	23 (20.4)				
≥60	82 (42.1)	68 (82.9)	14 (17.1)				
EGFR Mutation Status				0.001	0.001	3.036	1.557-5.922
Negative	112 (57.4)	98 (87.5)	14 (12.5)				
Positive	83 (42.6)	60 (72.3)	23 (27.7)				
Smoking				0.120	0.365		
No	102 (52.3)	78 (76.5)	24 (23.5)				
Yes	93 (47.7)	80 (86.0)	13 (14.0)				
T				0.584	0.476		
T1-2	108 (55.4)	88 (81.5)	20 (18.5)				
T3-4	87 (44.6)	70 (80.5)	17 (19.5)				
N				0.777	0.746		
N0-1	76 (39.0)	62 (81.6)	14 (18.4)				
N2-3	119 (61.0)	96 (80.7)	23 (19.3)				
Treatment before Subsequent BM							
CT				0.497	0.557		
No	35 (17.9)	28 (80.0)	7 (20.0)				
Yes	160 (82.1)	130 (81.3)	30 (18.8)				
RT				0.424	0.145		
No	137 (70.3)	113 (82.5)	24 (17.5)				
Yes	58 (29.7)	45 (77.6)	13 (22.4)				
S				0.975	0.433		
No	108 (55.4)	90 (83.3)	18 (16.7)				
Yes	87 (44.6)	68 (78.2)	19 (21.8)				
EGFR-TKIs				0.824	0.386		
No	150 (76.9)	121 (80.7)	29 (19.3)				
Yes	45 (23.1)	37 (82.2)	8 (17.8)				

Abbreviations: BM= brain metastases; CI= confidence interval; CT= chemotherapy; EGFR= epidermal growth factor receptor; EGFR-TKIs= epidermal growth factor receptor tyrosine kinase inhibitors; HR= hazard ratio; N= node; RT= radiotherapy for primary lesions of the lung; S= surgery treatment for primary lesions of the lung; T= tumor.

### EGFR-TKIs sensitive mutation and BM

Among various EGFR mutations, exon19 deletion and exon 21 L858R point mutation are EGFR TKI sensitive mutations. In order to compare BM incidences associated with these two mutations, subgroup analyses were performed in patients with exon 19 deletion (56 patients) and exon 21 L858R point mutation. The incidences of BM were not significantly different between these two subgroups of patients either at initial diagnosis (21.4% for exon 19 *vs*. 27.3% for exon 21, *P*=0.638) or at last follow-up (44.6% for exon 19 *vs*. 45.5% for exon 21, *P*=0.935). In addition, there were no between-subgroup differences in the 1-, 2- and 3-year accumulative rates of BM (18.5% for exon 19 *vs*. 23.3% for exon 21, 44.5% for exon 19 *vs*. 36.1% for exon 21, and 44.5% for exon 19 *vs*. 57.4% for exon 21; at 1, 2 and 3 years, respectively; *P*=0.619) (Figure 2B).

### OS of patients after diagnosis of BM with mutated and wild-type EGFR

**Figure 3:**
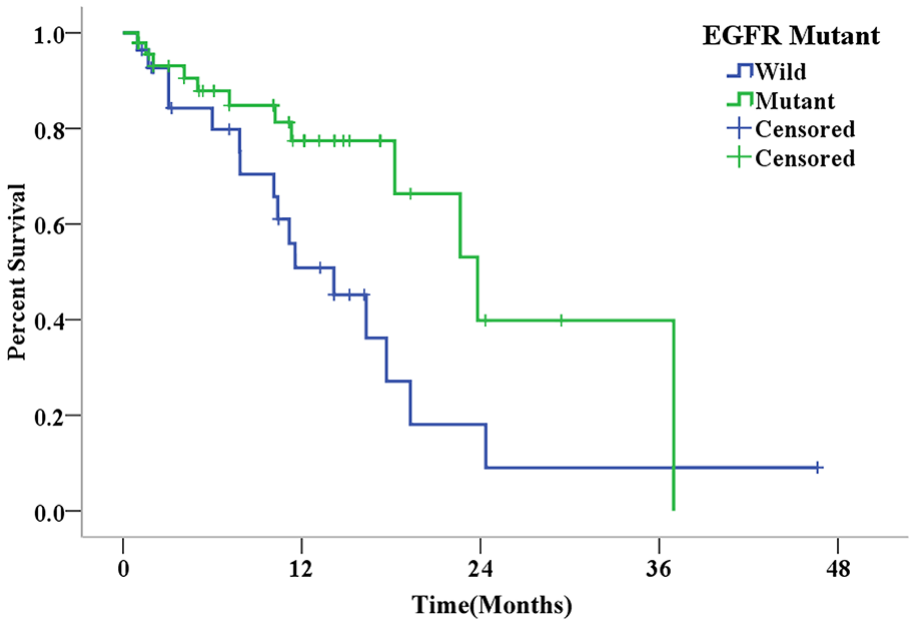
Kaplan-Meier curves for overall survival in patients with different EGFR mutation statuses after the diagnosis of final brain metastasis (*P*=0.028).

Seventy-six patients had final BM in this retrospective analysis. Table 5 shows the baseline characteristics of these patients stratified by EGFR mutation status (two groups). The rates of radiotherapy for BM were similar in both groups (*P*=0.704). However, chemotherapy was more frequently administered in the wild-type EGFR group than in the EGFR-mutant group (19/28 *vs*. 19/48, *P*=0.017), while EGFR-TKIs were more commonly administered in the EGFR-mutant group than in the wild-type EGFR group (21/48 *vs*. 2/28, *P*=0.001). There was a statistically significant difference in OS between the two groups (*P*=0.028). Furthermore, median OS was 23.8 months in the EGFR-mutant group (95% CI=17.13-30.47) and 14.2 months in the wild-type EGFR group (95% CI=8.55-19.79) (Figure 3).

**Table 5:** Characteristics of patients with finial BM stratified by EGFR mutation status (N=76)

Characteristics	Total (N =76)	EGFR Mutation Status		*P*
		Negative (%) (n=28)	Positive (%) (n=48)	
Gender				0.016
Male	30 (39.5)	16 (53.3)	14 (46.7)	
Female	46 (60.5)	12 (26.1)	34 (73.9)	
Age				0.645
<60	46 (60.5)	16 (34.8)	30 (65.2)	
≥60	30 (39.5)	12 (40.0)	18 (60.0)	
Smoking				0.068
No	53 (69.7)	16 (30.2)	37 (69.8)	
Yes	23 (30.3)	12 (52.2)	11 (47.8)	
ECOG PS				0.519
0-1	60 (78.9)	21 (35.0)	39 (65.0)	
2-4	16 (21.1)	7 (43.8)	9 (56.2)	
Treatment after BM				
RT				0.704
No	32 (42.1)	11 (34.4)	21 (65.6)	
Yes	44 (57.9)	17 (38.6)	27 (61.4)	
CT				0.017
No	38 (50.0)	9 (23.7)	29 (76.3)	
Yes	38 (50.0)	19 (50.0)	19 (50.0)	
EGFR-TKIs				0.001
No	53 (69.7)	26 (49.1)	27 (50.9)	
Yes	23 (30.3)	2 (8.7)	21 (91.3)	

Abbreviations: BM= brain metastases; CT= chemotherapy; ECOG PS= Eastern Cooperative Oncology Group Performance status; EGFR= epidermal growth factor receptor; EGFR-TKIs= epidermal growth factor receptor tyrosine kinase inhibitors; RT= radiotherapy for brain metastases.

## DISCUSSION

Adenocarcinoma is the most common NSCLC subtype. Studies have shown that adenocarcinoma histology is associated with a significantly higher incidence of BM than other subtypes of NSCLC [1, 23, 24]. In this study, we retrospectively evaluated the different features of BM according to EGFR mutation status in patients with pulmonary adenocarcinoma. Our results revealed that patients with EGFR-mutant tumors had a higher incidence of BM than patients with EGFR wild-type tumors both at the time of diagnosis and during the disease course. Brain metastases in patients with EGFR mutations were found to be larger and more diffuse than in patients with wild-type EGFR. To our knowledge, our research may represent the largest cohort study that analyzed the relationship between EGFR mutation status and BM in Chinese lung adenocarcinoma patients.

The frequency of EGFR mutation in pulmonary adenocarcinoma may vary in different ethnic populations. In East Asian patients, the reported EGFR mutation rate is 40%-60% [25]. In our study, EGFR mutation rate was 46.2%; and the most common mutations occurred at exon 19 and exon 21, which were similar to previously published data. Female pulmonary adenocarcinomas patients and never-smokers were reported to have a higher frequency of EGFR mutations [26], which was also in line with our study results (Table 1). The prevalence of EGFR mutation in Caucasians is less than 15% according to one report [27], which is much lower than that in the Asian population. The low prevalence of EGFR mutations in the Caucasian population may be the reason that some studies in non-Asian regions were unable to identify any difference in BM risk in patients with or without EGFR mutations [21, 22].

This is the first study to suggest that Chinese pulmonary adenocarcinoma patients with EGFR mutations may be more prone to the development of BM, but not ECMO, compared to patients with wild-type EGFR, both at initial diagnosis and during the course of the disease. Previous studies have shown the impact of EGFR mutation on BM, however, the potential relationship between EGFR mutation and extracranial metastases remains unreported [28, 29]. One study evaluated the impact of EGFR mutation on lung and bone metastases, and found that patients with EGFR mutations had elevated risks for lung and bone metastases [30]. The mechanism for BM may be different from that for metastases to other sites of the body. In order to successfully metastasize to the brain, tumor cells need to have special abilities to cross the blood brain barrier (BBB) and form colonies in the brain. Therefore, in this study, we distinguished all distant metastases without BM from BM, and classified these as ECMO. Future research should be directed to analyze the potential relationship between EGFR mutation status and ECMO to different organ sites.

Further analysis of data from patients with BM revealed that EGFR mutation was significantly associated with increased numbers and sizes of metastatic lesions in the brain. However, this was not consistent with the study conducted by Luo *et al* [29]. Brain imaging methods were not uniform in the study of Luo *et al*., while patients in our study all received routine brain MRI. This standardized approach minimized potential bias from using different methods for brain imaging.

Moreover, we demonstrated that there was no difference in BM rate between Chinese pulmonary adenocarcinoma patients with EGFR mutations in exon 19 and exon 21. However, another study reported different results. Li *et al*. [28] found that patients with EGFR mutations at exon 19 had the highest incidence of BM among patients with EGFR mutations. Selection bias and the small number of patients in the study conducted by Li *et al*. were possible causes for these differences. Therefore, it may be difficult to draw a conclusion from these data. Future clinical trials with large numbers of patients are needed to provide a definitive conclusion.

Although our study demonstrated the association between EGFR mutation and increased BM incidence, the exact underlying mechanisms remain unclear. Brenda *et al*. [31] discovered that EGFR signaling elevated C/EBPβ-LIP expression by increasing the binding activity of CUG-binding protein 1 (CUGBP1) to C/EBPβ mRNA; which may lead to the development of BM. Another study suggested that EGFR signaling may enhance cellular invasion ability mainly through the phosphoinositide 3-kinase/protein kinase B and phospholipase C γ downstream pathways, and EGFR inhibition significantly decreased BM *in vivo* [32]. Breindel *et al*. [33] reported that MET activation by EGFR signaling through mitogen-activated protein kinases (MAPK) promoted BM in NSCLC. EGFR mutation in lung cancer was often found to result in the activation of signal transducers and activators of transcription 3 (STAT3) [34, 35]. Recently, Singh *et al*. [36] identified STAT3 as an upregulator of lung-to-brain metastases. According to this study, the activation of the STAT3 signal pathway by EGFR mutation may contribute to increased BM risk for patients with EGFR mutations. Although these studies have provided some insights into the mechanisms underlying the increased BM risk associated with pulmonary adenocarcinomas with EGFR mutations, further investigations are needed to elucidate the exact role of EGFR in BM at the molecular level.

Previous studies have suggested that EFGR-TKIs treatment may be effective in delaying and/or preventing BM in NSCLC patients with EGFR mutations [37, 38]. However, in our study, EFGR-TKIs treatment was not significantly associated with a decreased risk of subsequent BM. This negative result may be attributed to the relatively small number of patients with EGFR mutations (30/83, 36.1%), who were treated with EFGR-TKIs prior to the development of subsequent BM. Further studies are warranted to clarify this issue. Prophylactic cranial irradiation (PCI) is a standard treatment for small cell lung cancer (SCLC) patients with proven effectiveness. However, in NSCLC patients, the use of PCI only reduced the cumulative incidence of BM, and did not improve OS [39]. This is in part due to differences in tumor biology and genetics across various pathological subtypes of NSCLC. It is perceived that only patients with higher risks of BM may benefit from PCI. Based on our findings, we hypothesize that PCI may also provide benefits for pulmonary adenocarcinoma patients with EGFR mutations (especially in exon 19 or 21), who cannot receive EGFR-TKIs for some reason. Well-designed prospective randomized clinical trials are warranted to validate our presupposition.

It was reported that EGFR mutation was associated with improved survival in NSCLC patients with BM [20]. Our study revealed similar results, in which EGFR mutation was a positive predictive factor for OS in Chinese pulmonary adenocarcinoma patients with BM. However, these results were contradictory to the findings of Lou *et al*. [29]. According to the study conducted by Lou *et al*., EGFR mutation status had no influence on progression free survival (PFS) or OS in Chinese NSCLC patients with BM (*n*=136). One possible explanation for this discrepancy is the use of EGFR-TKIs in patients with EGFR mutations, which may contribute to an improved OS. In the study of Lou *et al*., less than 10% of patients with EGFR mutations received EGFR-TKI treatment; while in our study, more than 40% of EGFR mutation patients with BM were treated with EGFR-TKIs. In several previous studies [9–11, 40], patients who received EGFR-TKIs at any time after the diagnosis of BM survived longer than patients who did not receive this treatment. In our study, EGFR-TKIs were administered more frequently in patients with BM and EGFR-mutant, compared with BM and wild-type EGFR; which may prolong OS.

There were some limitations in this study. First, this is a retrospective study, which may introduce potential bias resulting from uncontrolled factors involved in the complex treatment regimens such as treatment duration and concurrent therapy, since patients with lung adenocarcinoma received a wide variety of treatments. Second, the relatively low number of patients in this study may be insufficient to clearly define whether there is a strong link between EGFR mutations and BM. Third, the EGFR mutation status was evaluated by using samples from the original lung tumor rather than from the BM lesions, but the potential heterogeneity of tumor tissues was not taken into consideration in this study. Fourth, patients in this study did not receive periodic brain imaging scans; therefore the timing and incidence of BM may be inaccurate for asymptomatic patients. Fifth, since the neurological symptoms and deficit scores of patients were not available in the database, we were unable to evaluate the quality of life of patients with BM. Finally, this study did not evaluate the relationship between BM and other clinically relevant genetic changes such as KRAS mutation, ALK rearrangement, and MET amplification.

In conclusion, in this retrospective study, we have demonstrated that BM was more common among patients with EGFR-mutant lung adenocarcinoma (*vs*. wild-type EGFR lung adenocarcinoma). Thus, BM may represent as one of the distinct clinical features for EGFR-mutant tumors. EGFR mutation was shown to be an independent predictive and prognostic risk factor of BM for patients with lung adenocarcinoma, as well as a positive predictive factor for OS in patients with BM. Further molecular studies of EGFR-mutant tumors are needed to elucidate the mechanisms underlying this discovery.

## MATERIALS AND METHODS

### Patients

Inclusion criteria were as follows: (1) patients with pathologically confirmed lung adenocarcinoma, who underwent EGFR mutation screening and treatment at our institution between March 2007 and November 2014; (2) prior to the treatment, all patients who received initial staging work-up that consists of chest computed tomography (CT) scan, abdominal ultrasound/CT, bone scan and MRI of the brain; (3) the clinical stage was classified using the tumor, node, metastasis (TNM) system proposed by the American Joint Committee on Cancer (7^th^ edition). A total of 234 patients were included into this study. The study protocol was reviewed and approved by the Review Board and Ethics Committee of Hubei Cancer Hospital. This research was carried out in accordance with approved guidelines and the Declaration of Helsinki.

### Follow up and data collection

Patients were evaluated every three months for the first two years, then every 4-6 months for the next three years, and annually thereafter. Each follow-up evaluation consisted of history and physical examination, imaging studies including chest CT and abdominal ultrasound/CT, and other clinical examinations that were deemed necessary. Routine brain imaging was not performed during the follow-up period. Contrast-enhanced brain MRI scan was performed when BM was suspected. Disease progression and the sites of metastasis were determined by either imaging or histologic analysis, or both.

Clinical characteristics including age, gender, smoking history, treatment, time to progress to BM, the number of BM, the size of the largest metastatic brain lesion, survival time and so on, were obtained from medical records. Distant metastases were categorized as metastasis to the brain (patients with brain and extracranial metastases were included) or metastasis to extracranial sites only. BM at initial diagnosis was defined as “initial BM”, and BM found during and after treatment was recorded as “subsequent BM”. At the end of the follow-up, total BM (initial BM + subsequent BM) was documented as “final BM”. Similarly, according to the different timing of diagnosis, ECMO were recorded as “initial ECMO”, “subsequent ECMO” or “final ECMO”. The time to subsequent BM was calculated as the time between the date of the initial diagnosis and the date when BM was documented radiographically. The OS of patients after the diagnosis of final BM was calculated as the time between the date of BM diagnosis and the date of the last follow-up or death from any cause.

### EGFR mutation testing

**Figure 4:**
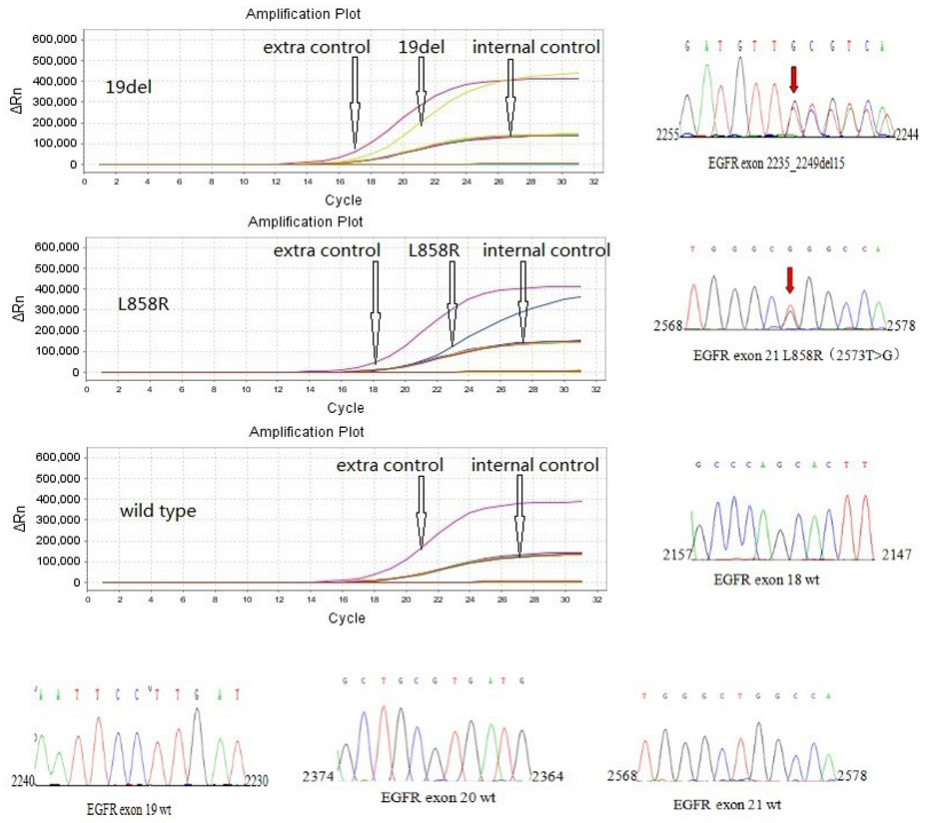
Representative results of EGFR mutation in exon19 (deletion) and exon 21 (L858R point mutation), and wild-type of EGFR using a HGN EGFR Mutations Detection Kit

EGFR gene mutations were analyzed in paraffin-embedded tissue sections from the primary tumor. Tumor tissue was scraped from glass slides under direct visualization or under a dissecting microscope, and Genomic DNA was extracted with a HGN-tq0850 DNA Kit (Hygeianey Bioscience Co. Ltd., Wuhan, China). EGFR mutations were detected using the HGN EGFR Mutations Detection Kit (Hygeianey Bioscience Co. Ltd., Wuhan, China) based on the principle of the peptide nucleic acid-locked nucleic acidpolymerase chain reaction clamp method [41]. The assay was carried out using the ABI 7500 (Applied Biosystems, Foster City, CA, USA) real-time polymerase chain reaction system, according to manufacturer's protocol. The representative results of the EGFR mutation test are shown in Figure 4.

### Statistical analysis

All data were processed with SPSS 19.0 (IBM SPSS Inc., Chicago, IL, USA). EGFR mutation status and BM frequency in different groups were compared using Chi-square test. Clinical factors known to be associated with initial BM were included in the logistic regression analysis. Univariate and multivariate analyses of risk factors for subsequent BM were performed using the log-rank test and Cox regression, respectively. The Kaplan-Meier method was used to calculate the time to subsequent BM and draw the survival curves. The number and the largest size of brain metastatic lesion in different EGFR mutation status groups were also compared using the Chi-square test. A 2-sided *P*<0.05 was considered statistically significant.
